# Capacity or Necessity? Comparing the Socio-Economic Distribution of Productive Activities Between Italy and South Korea

**DOI:** 10.1177/01640275221089203

**Published:** 2022-04-23

**Authors:** Ginevra Floridi

**Affiliations:** 1 Nuffield College and Leverhulme Centre for Demographic Science, 6396 University of Oxford, Oxford, UK

**Keywords:** employment, long-term care, inequality, socio-economic status, international comparative study

## Abstract

Much productive ageing research aims to identify the conditions under which older adults engage in productive roles within and outside the family. This study conceptualises two individual-level explanations for productive participation: *capacity* and *necessity*. I hypothesise that whether *capacity* or *necessity* prevails across different socio-economic groups depends on the degree of social protection guaranteed by pensions and long-term care systems, which varies across countries. Drawing on data from the SHARE and KLoSA surveys, this study compares socio-economic gradients in full-time work and informal caregiving across cohorts of men and women aged 50–75 in Italy and South Korea in 2006/07 and 2014/15. In Italy, where later-life social protection is generous, productive engagement is more common among wealthier and higher-educated individuals, who have greater capacity to engage in productive roles. In Korea, where social protection is limited, working is more common among socio-economically disadvantaged women, who have higher necessity to remain economically productive.

## Introduction

Many high-income countries currently face challenges with the financial sustainability of their pension and long-term care (LTC) systems in the face of population ageing (Colombo et al., 2011; OECD, 2019). In Southern Europe and East Asia, rapid and prolonged increases in the proportion of older adults in the population have prompted pension and LTC reforms over the last two decades (OECD, 2019; UN, 2019). The productive ageing framework has contributed to the policy discourse on ageing by emphasising that older adults remain ‘productive’ beyond retirement or the onset of disability (Bass & Caro, 1996). This framework highlights the importance of policies that facilitate and encourage older adults’ participation in activities that produce goods or services, or develop others’ capacity to do so, whether for pay or not (Bass & Caro, 2001). Productive ageing research investigates the conditions under which mid-life and older adults engage – or remain engaged – in productive roles, including paid work and informal caregiving for sick or disabled adults (Bass & Caro, 2001; Sherraden et al., 2001). Existing theoretical models point to individual and contextual determinants of participation in these roles (Sherraden et al., 2001). However, differences in the determinants of productive ageing across contexts have not been conceptualised.

In this study, I propose a distinction between two individual-level mechanisms behind productive ageing: *capacity* and *necessity*. *Individual capacity* entails a choice of whether to participate in productive roles based on one’s preferences and resources. *Necessity*, by contrast, relates to lack of choice as participation is mainly driven by the absence of affordable alternatives. I hypothesise that, at the country level, whether *capacity* or *necessity* prevails as the main motive behind productive engagement depends on the extent of social protection guaranteed to older adults. Later-life social protection is defined as the set of policies and programmes aimed at guaranteeing that older individuals can make a living without relying on their family members for financial or instrumental support. Pension and LTC systems represent the main components of social protection in later life (OECD, 2017).

To indirectly assess contextual variation in the importance of *capacity* and *necessity* as determinants of productive ageing, I compare socio-economic status (SES) gradients in paid work and informal caregiving among adults aged 50–75 between Italy and the Republic of Korea (Korea henceforth) in 2006/07 and 2014/15. Italy and Korea are interesting to compare because, while sharing important similarities in their welfare and labour market systems, they differ in the degree of social protection to older individuals (Estevez-Abe et al., 2016; Floridi, 2020). The comparison highlights existing SES inequalities in access and participation to productive roles, and how they vary across contexts. In Italy, where later-life protection is stronger, participation is higher among higher-SES individuals, who tend to have greater *capacity* to remain productive. In Korea, by contrast, participation is higher among lower-SES individuals, in line with their higher *necessity* to remain productive.

## Background

### Individual and Contextual Determinants of Productive Engagement

A starting point for understanding the determinants of productive engagement is the ‘productivity in later life’ model elaborated by Sherraden et al. (2001), which is based on the conceptual framework proposed by Bass and Caro (1996) (see Supplemental figure 1). Within this framework, socio-demographic characteristics including education, age and gender determine one’s *individual capacity* to participate in productive behaviours by shaping financial and time resources as well as physical and cognitive functioning. At the same time, contextual characteristics such as public policies and programmes determine the *institutional capacity* to participate, expressed by the number, type and quality of roles available (Sherraden et al., 2001). Combined, *individual* and *institutional capacity* influence participation in productive roles including market activities like paid work and non-market activities like informal caregiving for family members and friends, which have consequences for individuals, families, and communities (Sherraden et al., 2001).

A large body of empirical literature identifies the individual-level determinants of productive ageing. First, participation in the labour market and family care activities differs between men and women, in line with gendered socialisation into breadwinner and caregiver roles (Van der Meer, 2006). Younger and married individuals, those in better physical and cognitive health, and those engaged in reciprocal exchanges of financial and co-residential support with their family members are more likely to participate in productive roles such as working and caregiving. These results hold across countries as diverse as Vietnam (Giang et al., 2019), Finland (Akintayo et al., 2016) and Australia (Loh & Kendig, 2013). Productive participation also depends on family characteristics such as the presence of co-resident children, parents and grandchildren (Kim, 2020; Sabbath et al., 2016). Participation in paid work also differs by area of dwelling, with higher rates of inactivity in rural than urban areas (Van der Meer, 2006).

Regarding SES as a determinant of participation, studies in Western European countries, the United States (US) and Australia find that individuals with greater financial resources are more likely to work (Akintayo et al., 2016; Bukov et al., 2002; Loh & Kendig, 2013), and higher educational attainment is strongly linked with participation in productive roles including working and caregiving (Arpino & Solé-Auró, 2019; Kim, 2020). By contrast, a study of productive ageing in Vietnam (Giang et al., 2019) finds that individuals with no or little education are more likely than higher-educated individuals to participate in productive activities after age 50. Korean studies also find that, although older adults in paid work have higher incomes (Kim, 2019; Lee & Lee, 2014), education has a negative (Lee & Lee, 2014) or no (Kim, 2019; Kim et al., 2013) association with economic activity in mid- and later life. These findings suggest that SES variation in productive activities may be related to the institutional and cultural context where older adults live.

At the contextual level, participation in productive roles varies across institutional and cultural settings (Chen et al., 2016; Strauss & Trommer, 2018). Empirically, across Europe, paid work and informal caregiving in later life are more common in countries where social policies and programmes guarantee older adults’ ability to make a living independent of their families (Hank, 2011; Strauss & Trommer, 2018). A comparison of three South-East Asian countries shows that in Thailand, where economic development and protection to older adults are stronger than in Myanmar and Vietnam, participation in employment and family caregiving is higher, especially among higher-educated older adults (Teerawichitchainan et al., 2019).

### Socio-Economic Status, Capacity and Necessity

In reviewing the literature on the individual determinants of productive activities, it is interesting to note that, while socio-demographic, health and family characteristics are associated with productive activities in similar ways across different countries (Akintayo et al., 2016; Teerawichitchainan et al., 2019), the association of SES with productive ageing varies across contexts. As highlighted above, while higher-SES is linked with higher participation in Western countries, studies of East and Southeast Asian countries have found negative associations between SES and productive participation (Giang et al., 2019).

SES differences in productive ageing across contexts may partly relate to whether individual *capacity* or *necessity* prevails as the underlying motive behind participation. *Capacity* refers to an individual’s choice of whether to participate in productive roles, based on their preferences and resources. As such, *capacity* is intrinsically linked to having a minimum level of social and economic security. In contexts where pensions have high coverage and replacement rates, and LTC support is available in-cash or in-kind, individuals may in principle choose whether to participate or not in productive roles, as alternatives are available to earning money and providing care to disabled family members. As such, productive ageing may be more common among higher-SES individuals, who have higher *individual capacity* given by better job quality and career opportunities and higher resources facilitating the provision of informal care, such as own transportation (Arpino & Solé-Auró, 2019).

*Individual necessity*, on the other hand, entails little choice, with participation driven by lack of suitable alternatives. *Necessity* is strongly related to lack of social protection, such as in the case of limited pension and LTC coverage. *Necessity* may be particularly strong for socio-economically disadvantaged older adults, who lack alternative options to earning money by working, or to providing care to disabled relatives (Giang et al., 2019). So far, differences between contexts with varying levels of social protection to older adults have not been conceptualised, as there are few cross-national comparative studies on the individual predictors of productive ageing (Teerawichitchainan et al., 2019).

**Figure 1.**
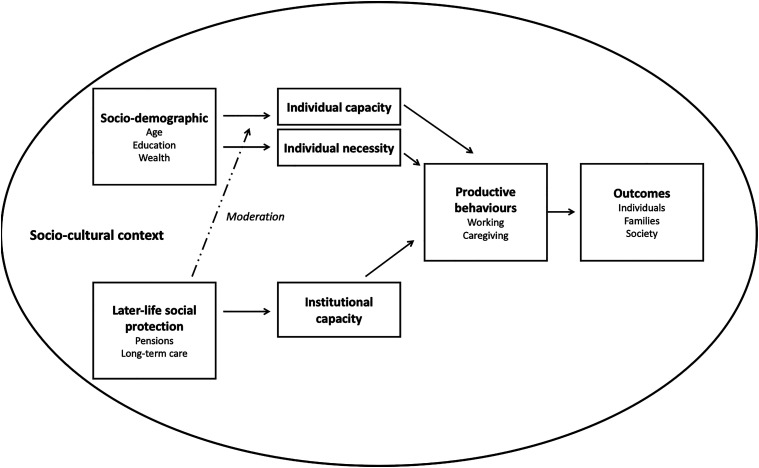
Conceptual framework.

The conceptual model proposed here, adapted from Sherraden et al. (2001) and summarised in Figure 1, incorporates interactions between contextual and individual determinants to study how later-life social protection guaranteed by pension and LTC systems moderates the association between individual characteristics (including SES) and productive participation. In the model, later-life social protection influences productive activities directly by affecting *institutional capacity*. At the same time, it moderates the association between individual characteristics (such as SES) and productive activities. Moderation occurs because social protection shapes the presence of alternatives to paid work and family care, and in turn the degree to which *capacity* or *necessity* prevail as motives behind those activities.

The model adds to existing conceptualisations of the determinants of productive ageing by distinguishing between *individual capacity* and *necessity*. Among older adults’ individual characteristics, I take SES as a proxy for individual resources (*capacity*) and need (*necessity*). Higher – as opposed to lower – SES enables individuals to access better jobs, forfeit work in favour of leisure, or pay for care instead of directly providing it. By contrast, low SES may partly coerce individuals into productive roles, especially in contexts where alternatives to paid work or family care are scarce. Thus, while *individual capacity* (proxied by higher-SES) may prevail as a motive for participation in countries where a basic level of social protection to older adults is guaranteed, *individual necessity* (proxied by lower-SES) may be more relevant in contexts of low social protection. An indirect test of these mechanisms may be provided by comparing SES gradients in productive activities between two countries with different levels of social protection to older adults: Italy and Korea.

### Italy and Korea

Italy and Korea are among the fastest-ageing countries in the world (UN, 2019) and have comparable levels of economic development. Despite their geographic distance, they share affinities in social policies and labour market features that have led some authors to classify them as belonging to the same ‘family of nations’ (Estevez-Abe et al., 2016). Four main characteristics summarise these similarities: (*i)* familistic welfare models, in the sense that families are assumed to be responsible for much of the financial and care support to their dependent members; (*ii)* labour markets characterised by a distinction between the formal and informal sectors, with little mobility between the two; (*iii)* welfare benefits tied to working in the formal sector, with informal and temporary workers and non-employed individuals excluded from coverage; and (*iv)* welfare systems originally based on a male-breadwinner model by which men work and provide financial support, and women provide informal care to children and older or disabled adults (Estevez-Abe et al., 2016; Saraceno, 2016). In both countries, there are large differences in working and caregiving rates between men and women, due to gendered divisions of labour that remain pronounced among older age groups (Floridi, 2020; Saraceno, 2016).

Notwithstanding their similarities, Italy and Korea are characterised by important differences in their later-life social protection through pension and LTC systems. Table 1 summarises the main characteristics of each country in relation to pension and LTC systems in 2006/07 and 2014/15, which correspond to the two observation points for this study.

**Table 1.** Comparison of pension and old-age benefits, long-term care and childcare systems between Italy and South Korea (2005–2015).

	Italy		South Korea	
	2006/7	2014/15	2006/7	2014/15
Pensions and Old-Age Benefits				
Main pillars	Public pensions	Public pensions	National Pension System (NPS) + private pensions	National Pension System (NPS) + private pensions + old-age security benefits
Coverage of over-65	Nearly universal coverage of over-65	Nearly universal coverage of over-65	NPS + old-age security benefits: Around 30% of over-65	NPS + old-age security benefits: 70% of over-65
Public spending on pensions and old-age benefits as % GDP	13.7% (OECD average 6.8%)	16.2% (OECD average 8.0%)	1.5% (OECD average 6.8%)	2.9% (OECD average 8.0%)
Net public pension replacement rate, % pre-retirement earnings (average earner)	79%	80%	41%	45%
Normal retirement age for someone who entered the labour market at age 20	60 (women) 65 (men)	65.6 (women) 66.6 (men)	60 (women) 60 (men)	61 (women) 61 (men)
Average effective age of exit from the labour market	58.7 (women) 62.0 (men)	61 (women) 61.5 (men)	67.5 (women) 70.9 (men)	70 (women) 73 (men)
Employment rate for 55–64-year-olds	32.42%	46.23%	58.36%	65.75%
Old-age security (state pensions and other benefits)	Tax-exempt pension supplement of around €4725 per year for those with contributory pension below minimum level. Benefits increase to around €6700 per year from age 70	Tax-exempt pension supplement of around €5800 per year for those with contributory pension below minimum level. Benefits increase to around €8300 per year from age 70	Basic Livelihood Security System/Old Age Allowance: Around 5% average earnings from the NPS for individuals with low/no income and no person under obligation to support	Basic pension: Around 10% average earnings from NPS covering 70% of 65+; Basic livelihood: (Housing/medical benefits): Only if no person under obligation to support
Long-term care (LTC)				
Main pillars	Attendance allowance: Cash benefit of around €440 per month independent of income; services provided by municipalities	Attendance allowance: Cash benefit of around €500 per month independent of income; services provided by municipalities	No public long-term care insurance system until 2008	LTC insurance system: Providing benefits for (market-based) home and residential services
Coverage (public LTC recipients at home or institutions)	3% of over-65	5.5% of over-65	2.5% of over-65%	7.4% of over-65
Public spending on LTC as % of GDP	0.6% (OECD average 0.8%)	0.7% (OECD average 1.1%)	0.1% (OECD average 0.8%)	0.8% (OECD average 1.1%)
Adult care leave	Care leave granted to public and private employees who care for severely disabled relatives. Beneficiaries can expect 3 working days of paid leave (at 100% of the last salary) per month; and up to 2 years of paid leave. In addition, it is fully compensated and receives pension coverage	Care leave granted to public and private employees who care for severely disabled relatives. Beneficiaries can expect 3 working days of paid leave (at 100% of the last salary) per month; and up to 2 years of paid leave. In addition, it is fully compensated and receives pension coverage	Unpaid family care leave for up to 90 days per year. Can only be requested by employees. This leave may be taken in increments, as long as one increment is at least 30 days	Unpaid family care leave for up to 90 days per year. Can only be requested by employees. This leave may be taken in increments, as long as one increment is at least 30 days

Data sources: OECD (2021a) Social Expenditure Database (SOCX); OECD (2021b) Health expenditure and financing; OECD (2021c) Labour force statistics.

In Italy, familism is the result of institutional inertia and the slow adaptation of policies to socio-demographic changes such as ageing and rising female employment (Pavolini et al., 2016). Italians born before the 1960s have benefitted from employment growth and long-tenure jobs in the formal sector during their lifetime. Labour market liberalisation since the 1990s has predominantly affected newcomers, leaving younger individuals disproportionately exposed to unemployment and informal employment in the wake of the 2008 financial crisis (Jin et al., 2016). Public financial support is generous towards older age groups, as exemplified by nearly universal coverage of public pensions, high average replacement rates and cash benefits to low-income pensioners (Table 1). Over the study period, a pension reform in 2011 raised retirement ages, introduced penalties for early exit and set up a new pension calculator based on the contributions to the system over the working life rather than on the salary received in the last years of employment. Notably, these changes have mainly increased employment rates individuals born in the 1960s or afterwards (Jin et al., 2016).

Between 2005 and 2015 Korea has, by contrast, the highest old-age poverty rate in the OECD (2017). Pensions and old-age benefits are underdeveloped, with non-contributory pensions often contingent, by law, on the absence of support from family members (Table 1). The employment rate among older adults is high, and individuals born before the 1960s are overrepresented in the agricultural and informal service sectors, where many continue working long after reaching pensionable age (Table 1). At the same time, pension receipt is strongly tied to having worked in the formal sector (Jones & Fukawa, 2016).

Regarding LTC policy, in Italy institutional inertia is evident in the lack of substantial policy change between 2006/07 and 2014/15 (Table 1). Support for adults with care needs is provided in the form of cash transfers – the so-called ‘attendance allowance’ – with levels of LTC service provision varying widely across regions (Pavolini et al., 2016; Saraceno, 2016). The relative generosity of the cash allowance and the lack of restrictions on how it should be spent protect the financial security of disabled older adults and their families while fuelling a ‘migrant care worker’ model of care (Pavolini et al., 2016). Workers who have relatives with care needs can also benefit from relatively generous care leave schemes, with full salaries paid for up to 3 days per month.

Between 2006/07 and 2014/15 Korea represents, by contrast, a LTC system in transition. As shown in Table 1, the coverage of subsidised LTC services has considerably expanded over this period. Since 2008, a LTC insurance system has funded the expansion of market-based LTC. However, the high share of costs to be borne by beneficiaries (around 15–20% of the total) excludes the poorest from coverage, and formal LTC remains unaffordable for families at the bottom of the wealth distribution (Chon, 2014). Over the period considered, family care leave options have remained limited, with unpaid care leave for employees available to take in monthly instalments, involving substantial loss of income for caregivers.

The comparison highlights important policy differences between the two contexts. The first concerns the level of social protection to older adults between the mid-2000s and the mid-2010s (Floridi, 2020). In Italy, public policies and programs have safeguarded older individuals financially, through pensions and cash transfers, despite the scarce direct provision of care services. The Korean system, by contrast, has remained relatively limited in providing aid to financially insecure older individuals (Jones & Fukawa, 2016), focussing more on the subsidisation of services. The second difference lies in the degree of policy change as opposed to inertia (Saraceno, 2016). While in Italy there has been little change over the observation period except for increasing retirement ages, the Korean LTC and pension systems have been transformed to provide increased social protection.

Population ageing, labour market characteristics and the similar (gendered) models of welfare familism make the two countries comparable with respect to older adults’ participation in employment and family care (Floridi, 2020). At the same time, the contrasting levels of later-life social protection suggest that differences in the socio-economic distribution of paid work and informal caregiving may be studied to understand the prevailing mechanisms behind productive participation. Specifically, *individual capacity* may be the prevailing motive behind productive ageing in Italy (and higher among higher-SES groups); while *necessity* may be more relevant in Korea (and higher among lower-SES groups). A description of cultural similarities and differences between the two countries is beyond the scope of this study. However, analyses of attitudinal and values surveys indicate that both Italy and Korea display high levels of agreement about the importance of family obligations, low agreement with individualistic values and relatively unequal gender roles (Arpino & Tavares, 2013; Iwai & Yasuda, 2011). These suggest that, while both paid work and family care are productive accomplishments, the former may be normative for older men and the latter for older women.

### Study Objectives and Hypotheses

In this study, I empirically compare SES differences in paid work and caregiving to sick or disabled adults among cohorts of men and women aged 50–75 in Italy and Korea in 2006/07 and 2014/15. Specifically, I ask:

A. How is SES associated with participation in paid work and informal caregiving among individuals aged 50–75 in Italy and South Korea?

B. How did SES gradients in paid work and informal caregiving change in each country between 2006/07 and 2014/15?

The analysis is restricted to the 50–75 age group because most individuals in each country stop working within this age window (OECD, 2019), and are unlikely to provide informal care after 75 due to the physically demanding nature of personal care tasks. I use equivalised wealth and education as measures of SES. In contrast with income, these are not directly tied to employment, and can be studied as predictors of later-life work. Relative to income, wealth and education are more strongly related to older adults’ life courses, reflecting cumulative advantages and disadvantages (Robert & House, 1996). Education is mostly completed before age 50, and wealth takes into account lifetime accumulation of savings and assets.

I formulate two hypotheses about the socio-economic distribution of productive activities:

H1: In Italy, net of other characteristics, (a) participation in paid work and informal caregiving is more common among higher- (relative to lower-) SES individuals, and (b) SES gradients remain stable over time.

H2: In Korea, net of other characteristics, (a) participation in paid work and informal caregiving is more common among lower- (relative to higher-) SES individuals, and (b) SES differences decrease between 2006/07 and 2014/15.

In Italy, relatively generous old-age social protection has remained stable over time, such that higher *individual capacity* (higher SES) is expected to relate to greater productive participation with no or little change over time. In Korea, where social protection is limited, *individual necessity* (lower SES) may be more important as a determinant of participation, but existing inequalities may have decreased over time in line with policy reform.

## Research Design

### Data and Samples Specification

I used data from the Italian sample of the Survey of Health, Ageing and Retirement in Europe (SHARE) and from the Korean Longitudinal Study of Ageing (KLoSA). Both datasets are part of a family of harmonised longitudinal ageing surveys (see https://g2aging.org/). The target population of SHARE Italy included community-dwelling individuals aged 50 and above, and their spouses regardless of age (Börsch-Supan & Jürges, 2005). KLoSA targeted instead the population aged 45 and above, excluding younger spouses and people living in institutions at baseline (KLI, 2007).

At the time of writing, the datasets covered the period 2004–2020 for Italy (SHARE waves 1–8) and 2006–2018 for Korea (KLoSA waves 1–7). SHARE Italy has a baseline sample size of 2558 individuals, augmented by refreshment samples in subsequent waves. The baseline sample of KLoSA consists of 10,248 respondents, with a refreshment sample in wave 5 (2014). I used two waves of each survey, namely SHARE waves 2 (2006/7) and 6 (2015) and KLoSA waves 1 (2006) and 5 (2014). For KLoSA, these waves are well representative of the 50–75 population (as wave 5 includes a refreshment sample). For SHARE, these are the closest in time to KLoSA waves, in which it is possible to distinguish the provision of personal care (one of the outcomes of the analysis) from household help. For each survey, I treated each wave as an independent cross-section. This is because, rather than being interested in changes in the association between SES and productive activities within individuals over time, I am interested in how SES gradients in participation differ between Italy and Korea at each point in time (research question A), as well as between cohorts of adults aged 50–75 in 2006/07 and 2014/15 in each country (research question B). Around 20% of Italian and 40% of Korean sample respondents appeared in both waves of each survey. Excluding those respondents from the analysis did not substantively change the results. Moreover, to check for representativeness of the results with respect to the 50–75 populations of Italy and Korea in each year, I performed sensitivity analysis using the calibrated cross-sectional weights provided by both surveys.

In line with the gendered nature of working and caregiving participation in both countries (Saraceno, 2016), I conducted all analyses separately for men and women, and restricted the samples to respondents aged 50–75 at the time of the interview with no missing information on the variables of interest. The final samples consisted of 2062 Italians in 2006/07 (953 men and 1109 women); 6413 Koreans in 2006 (2969 men and 3444 women); 3603 Italians in 2015 (1627 men and 1976 women); and 5469 Koreans in 2014 (2407 men and 3062 women).

### Measures

*Dependent variables.* I classified respondents as working full-time if, at the time of the interview, they worked for pay for at least 30 hours per week. This includes employed and self-employed workers as well as those working for a family business. Anyone not working for at least 30 hours per week was classified as ‘not working full-time’. For informal caregiving, I coded a binary indicator of whether, in the last 12 months, respondents provided personal care at least once per week to any family members or friends, including spouses, parents, other relatives and friends. I restricted the definition of care to helping relatives or friends with Activities of Daily Living (ADL) or Instrumental Activities of Daily Living (IADL)^1^.

*Socio-economic status*. Indicators of SES are wealth and education as outlined above. I coded wealth as equivalised net worth, including assets (financial and real) minus all liabilities. In SHARE, this is reported at household level, and equivalised following Hagenaars et al. (1994) by assigning value 1 to the respondent, value 0.5 to each additional adult, and 0.3 to each child. In KLoSA, wealth is coded at individual or couple level, and equivalised using the same criterion (with value 0.5 assigned partners, if present). For comparability, wealth was split into five equal quintile groups, constructed separately by country and period of observation, with each containing 20% of the sample. For education, I coded a binary indicator of whether respondents had attained higher (i.e. secondary or higher) education or not.

*Control variables*. I coded a set of control variables all found to be relevant predictors of productive ageing in previous literature (Akintayo et al., 2016; Sabbath et al., 2016; Teerawichitchainan et al., 2019). These include age, marital status, rural (vs. urban) dwelling, presence of limitations with ADL or IADL, self-rated health and cognitive function. I coded family characteristics including child co-residence status, the presence of parents or grandchildren, and the receipt and giving of financial support any family member or friend (Giang et al., 2019). Finally, each of the dependent variables (working and caregiving) were included as covariates in the model for the other. In addition to the individual-level variables above, I coded indicators for country (Italy or Korea) and time of observation (2006/07 or 2014/15), which I used to test for differences in the SES gradients between the two countries and over time. Supplemental Table 1 reports the detailed coding of all variables in the study.

### Statistical Analysis

I regressed the binary indicators for paid work and informal caregiving on the full set of individual-level control variables described above using logistic regression, pooled by country and separately by gender. The final model specification was guided by exploratory analysis, as follows. First, to determine which associations differed significantly between the two countries – and should be modelled as such – I fitted logistic regression models for each outcome on the pooled male and female datasets separately by observation period, interacting all independent variables with a country indicator (see Supplemental Table 2). Since many coefficients including those on education, wealth, age, health and family characteristics differed significantly between the two countries (as indicated by significant interaction terms), it was deemed more appropriate to fit separate models by country. Second, separate models by country with all covariates interacted with an indicator for observation time (2006/07 vs. 2014/15) indicated that, except for SES and age, associations between individual characteristics and each productive activity remained relatively stable over time for men and women within the same country, with few exceptions (see Supplemental Table 3). Therefore, the final specification consisted of separate models by country for men and women, where SES and age were interacted with an indicator for observation time (2006/07 vs. 2014/15) to account for temporal changes in their associations with working and caregiving. I report unweighted results from these models and perform sensitivity to using the cross-sectional survey weights provided by SHARE and KLoSA as probability weights (Solon et al., 2015). The weights were rescaled by the relative size of the 50+ population by year and country (UN, 2019). All analysis were carried out using Stata 16.

## Results

### Descriptive Sample Characteristics

Table 2 provides summary measures for all the dependent and independent variables with respect to the analytic samples of 50–75-year-olds identified by country, observation period and gender.

**Table 2.** Descriptive sample characteristics by country, observation period and gender.

	Italy 2006/7		Italy 2015		Korea 2006		Korea 2014	
	Men	Women	Men	Women	Men	Women	Men	Women
Work 30+ hours (%)	24.34	10.55	33.50	19.99	51.43	19.98	63.98	34.13
Informal care provision (%)	13.12	23.78	10.26	18.37	2.25	3.54	1.41	1.63
Secondary+ education (%)	56.35	45.00	69.21	63.31	67.53	39.14	81.80	61.33
Wealth quintile: 1^st^ (%)	17.84	19.03	18.01	18.52	16.67	21.52	13.05	19.69
2^nd^	21.20	20.29	21.39	20.29	18.39	20.62	18.11	19.17
3^rd^	21.30	19.57	18.68	20.19	20.82	21.02	21.23	19.60
4^th^	20.15	20.20	21.45	20.95	21.52	18.87	23.02	20.77
5^th^	19.52	20.92	20.47	20.04	22.60	17.97	24.59	20.77
Age (mean)	63.47	61.87	63.66	62.56	61.68	61.48	62.21	61.83
Marital status: Married (%)	94.33	84.04	87.43	97.25	94.24	74.42	94.14	79.49
Widowed	3.25	13.17	2.09	9.87	3.47	22.79	2.87	16.85
Not married	2.41	2.80	10.57	10.88	2.29	2.79	2.99	3.66
Rural (vs. urban) area	44.39	44.09	27.97	29.55	24.12	24.62	21.27	22.83
Any ADL limitations (%)	4.93	7.60	3.75	5.26	2.51	2.15	1.93	1.20
Any IADL limitations (%)	9.55	15.91	4.55	8.20	12.54	6.24	8.85	1.76
Self-rated health: Excellent (%)	8.81	6.40	7.81	6.68	4.08	2.15	1.41	0.00
Very good	14.17	14.16	18.13	16.90	42.81	27.44	8.39	6.37
Good	42.08	35.17	42.90	38.97	32.37	33.22	48.61	41.28
Fair	25.50	34.54	26.43	31.33	17.35	30.14	31.99	37.17
Poor	9.44	9.74	4.73	6.12	3.40	7.06	9.60	15.19
Cognition score (mean)	3.83	3.87	3.92	3.89	3.83	3.67	3.88	3.83
Parent status: Childless (%)	10.36	10.16	12.97	12.96	1.68	2.26	3.38	2.29
All children outside household	42.95	46.41	39.77	43.47	46.99	51.02	48.44	53.19
Any co-resident children	46.70	43.12	47.26	43.57	51.33	46.72	48.18	44.52
Either parent alive (%)	26.23	28.49	29.01	34.77	28.46	24.91	35.60	33.83
Any grandchildren (%)	58.34	63.12	47.20	54.50	59.08	72.07	54.30	63.42
Giving financial support (%)	41.03	39.86	30.24	29.50	26.34	19.37	37.56	30.11
Receiving financial support (%)	7.87	8.12	5.65	6.58	41.56	51.16	47.69	57.12
N. Individuals	953	1109	1627	1976	2969	3444	2407	3062

In both countries, and for both genders, adults aged 50–75 are more likely to work in 2014/15 than in 2006/07. Notably, at any time point, Korean men are more likely work for pay for 30+ hours than Italian men (51–64% as opposed to 24–34%), while Korean women are more likely to work than Italian women (20–34% as opposed to 10–20%). The opposite is true for informal care provision to relatives and friends. Informal caregiving to sick or disabled adults decreases in both countries between 2006/07 and 2014/15. In Italy, around 13–10% of men and 24–18% of women provide personal care, while in Korea the corresponding figures are 2–1% and 2–4%, respectively. Previous studies have documented similarly low proportions of older Koreans engaged in informal caregiving (Do, 2008; Do et al., 2015). A potential explanation is that, in Korea, the primary caregivers for sick or disabled individuals are traditionally daughters or daughters-in-law (Do et al., 2015; Kong, 2007). While differences in respondents’ interpretation of the caregiving questions between SHARE and KLoSA may also play a role, this is unlikely to be important, since both surveys show respondents detailed lists of what tasks count as personal care (e.g. help with bathing and eating)^1^ (Börsch-Supan & Jürges, 2005; KLI, 2007).

Korean men are more likely to have secondary or higher education than Italian men, while rates are similar for women indicating greater gender disparities in educational attainment in Korea. Overall, Italian respondents in 2006/07 are more likely than Koreans to live in rural areas or villages, but differences decrease by 2014/15. Some differences emerge in self-reports of health, with Italian respondents more likely to select ‘good’ or ‘fair’ health at any time point, and Koreans more likely to report ‘very good’, ‘good’ or ‘fair’ health in 2006/7 and ‘good’ or ‘fair’ health in 2014. Those differences may be driven by cultural perceptions of health as well as differences in objective health status (Jürges, 2007), and provide further justification for the separate modelling by country. Family characteristics differ between the two samples, with Italian respondents more likely to be childless and Korean respondents more likely to have grandchildren. There are large differences in the probability of giving and receiving financial support to family and friends, as Italian men and women are more likely to give and less likely to receive informal financial gifts than Koreans. These are in line with previous research showing that financial support flows mainly upwards (from children to parents) in Korea, and mainly downwards (from parents to children) in Italy (Floridi, 2020).

### Comparing Socio-Economic Gradients in Paid Work

**Figure 2.**
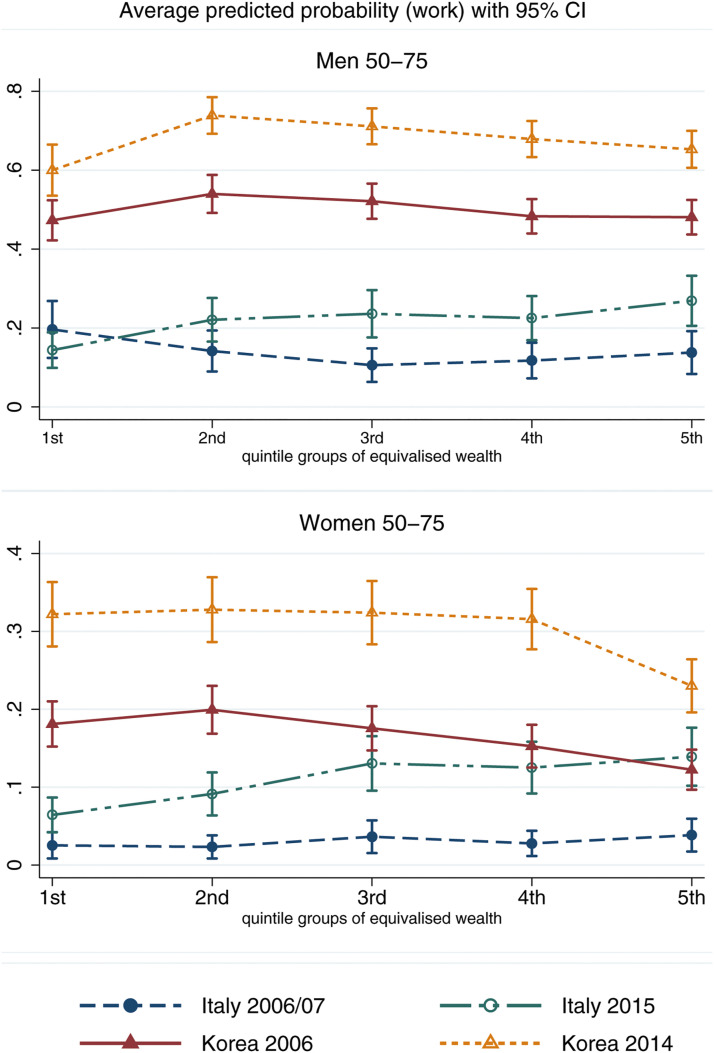
Average predicted probability of full-time paid work with 95% confidence interval, calculated at different quintile groups of equivalised wealth, by gender. The values of all other covariates in the model are fixed at their mean values.

**Figure 3.**
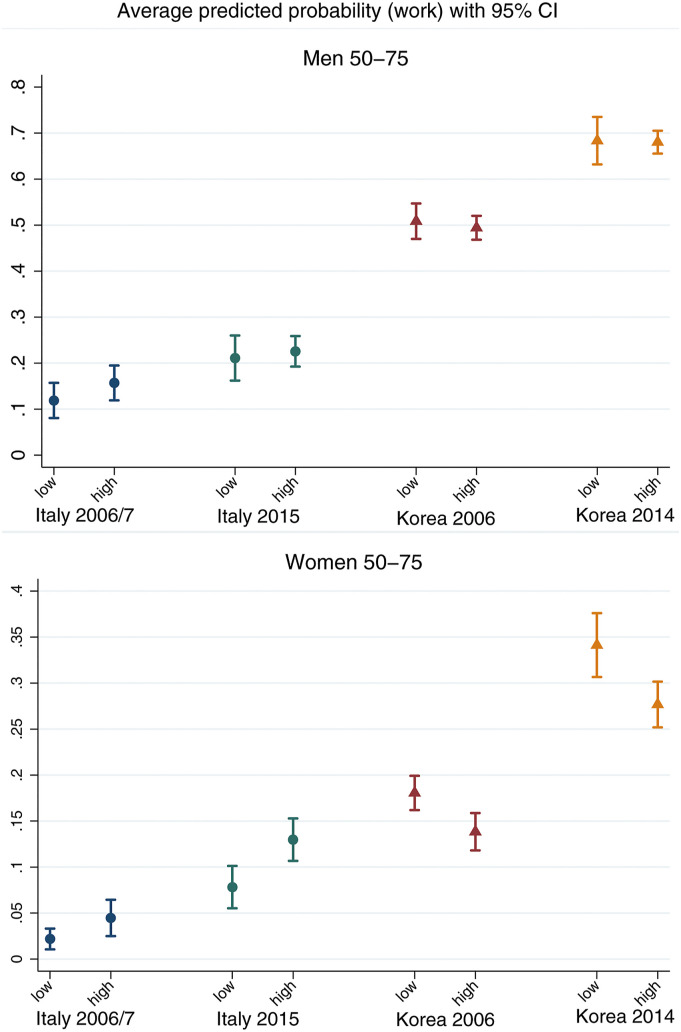
Average predicted probability of full-time paid work with 95% confidence interval, calculated at different levels of educational attainment, by gender. The values of all other covariates in the model are fixed at their mean values.

Figures 2 and 3 report the average predicted probability of working for 30+ hours per week by groups of equivalised wealth (Figure 2) and educational attainment (Figure 3), by country-time and separately for men and women. These represent the average probabilities of working predicted by the model for individuals with control variables fixed at their sample means. 95% confidence intervals are reported to evaluate the statistical significance of socio-economic differences in participation. The corresponding model coefficients are reported in Supplemental Table 4.

The top panel of Figure 2 shows that, in 2006/07, there are no statistically significant differences in paid work participation by wealth in either country, although Italian men in the middle of the wealth distribution are somewhat less likely to work than those at the bottom. By 2015, there is a statistically significant positive wealth gradient in paid work participation among Italian men, with those at the top around 10 percentage points (p.p.) more likely to work than those at the bottom of the wealth distribution. In Korea, by contrast, men in the second poorest group are around 10–15p.p. more likely to work full-time than those at the top and bottom of the wealth distribution. The results for Italian women (Figure 2, bottom panel) show no wealth gradients in paid work participation in 2006/07. In 2015, however, Italian women in the three wealthiest groups are significantly more likely to work than those at the bottom by around 8p.p, mirroring the gradient found for Italian men. By contrast, among Korean women, those in the top 20% of the wealth distribution are significantly less likely to work than other groups, with differences around 7–8p.p., and slightly larger in 2014 relative to 2006.

Figure 3 shows the predicted probability of working full-time for individuals with no or primary (‘low’) as opposed to secondary or higher (‘high’) education. The top panel indicates that there are no statistical differences in paid work participation by educational attainment for men in either country. For women (Figure 2, bottom panel), stark differences emerge: in Italy, those with higher education are significantly more likely to work than those with lower education in 2015 by about 6p.p. In Korea, higher-educated women are significantly less likely to work full-time in both observation periods by 6–7p.p., confirming the gradient found with respect to wealth. The findings are largely in line with hypotheses *H1(a)* and *H2(a)*, as lower SES is associated with lower paid work participation in Italy, but higher participation in Korea (with the only exception of Korean men in the bottom 20% of the wealth distribution). *H1(b)* and *H2(b)* are not confirmed for paid work, as differences in the SES gradient in participation become larger in Italy and remain relatively stable in Korea between 2006/07 and 2014/15.

### Comparing Socio-Economic Gradients in Informal Caregiving

**Figure 4.**
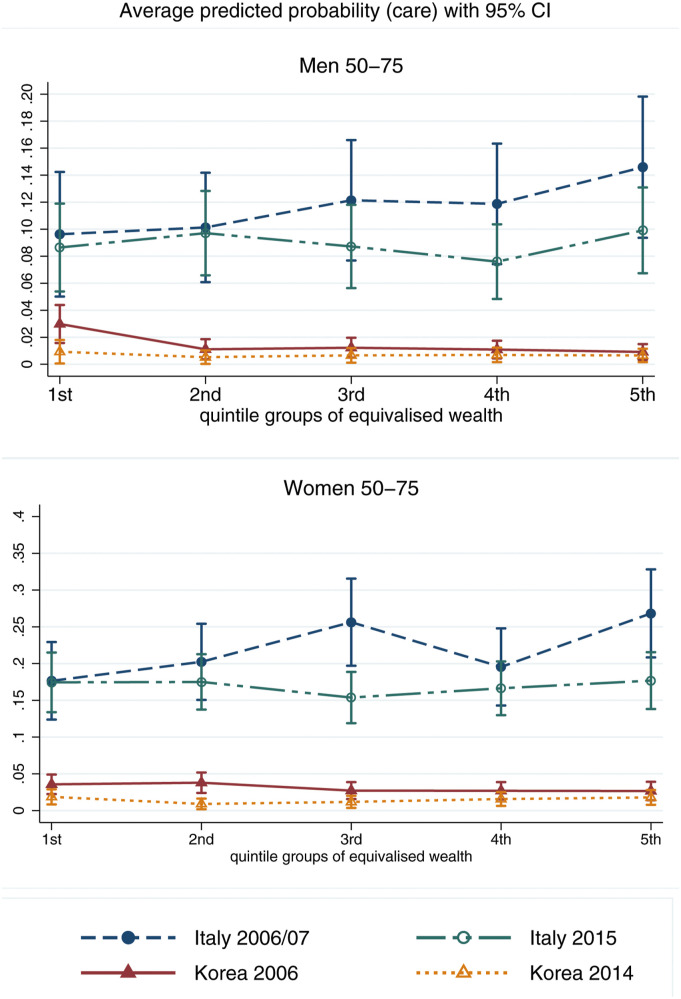
Average predicted probability of caregiving for sick or disabled adults with 95% confidence interval, calculated at different quintile groups of equivalised wealth, by gender. The values of all other covariates in the model are fixed at their mean values.

**Figure 5.**
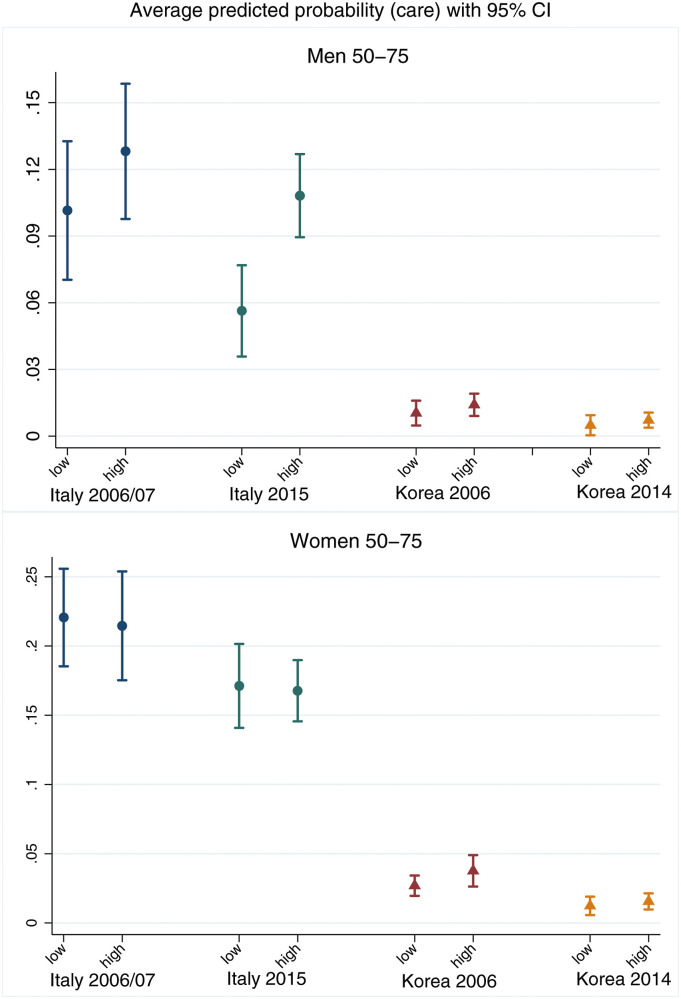
Average predicted probability of caregiving for sick or disabled adults with 95% confidence interval, calculated at different levels of educational attainment, by gender. The values of all other covariates in the model are fixed at their mean values.

Figures 4 and 5 report the wealth and education gradients in informal care provision for sick or disabled family members or friends, with corresponding 95% confidence intervals. Supplemental Table 5 reports the corresponding model coefficients.

For men (Figure 4, top panel), there is no significant wealth gradient in informal care provision in Italy at either observation point. In Korea, men at the bottom of the wealth distribution are significantly more likely to provide informal care in 2006 by 2p.p., a large difference considering the overall probability of providing care for Korean men in 2006 is 2.3% (Table 2). This gradient becomes null in 2014. For women (Figure 4, bottom panel), in Italy those in the middle and top of the wealth distribution are significantly more likely to provide care by about 8p.p., but this is only true in 2006/07 with no statistically relevant gradient found in 2015. The figure shows no differences in the probability of caregiving by wealth for Korean women in either period.

When looking at differences in informal care provision by educational attainment (Figure 5), higher-educated Italian men are significantly more likely to provide care in 2015 than those with lower education by 5p.p. For Korean men, there are no statistical differences in informal care provision by educational attainment (Figure 5, top panel). For women (Figure 5, bottom panel), none of the differences in informal care provision by education reach conventional levels of statistical significance. Overall, these results lend some weak support to *H1(a)* and *H2(a)*, with positive wealth gradients in Italy for women in 2006/07 and positive educational gradients for men in 2015, and negative wealth gradients for Korean men in 2006. While for Korean men SES gradients in care participation decrease by 2014/15 compared to 2006/07, lending some support to *H2(b)*, the results do not confirm *H1(b)* for Italy.

### Sensitivity to Standardised Calibrated Cross-Sectional Weights

All results are confirmed when using survey weights (Supplemental figures 2-5), except for the wealth gradient in paid work among Italian women in 2015, which becomes non-statistically significant despite maintaining the same direction. This does not change the interpretation of results overall as the educational gradient for Italian women in paid work remains in favour of the highly educated in 2015, in sharp contrast to the educational gradient for Korean women.

## Discussion and Conclusion

This paper adds to the literature on productive ageing by proposing a distinction between two individual motives behind productive engagement: *capacity* and *necessity*. I argue that later-life social protection in a country may act to influence the degree to which older adults of different SES may choose whether to engage in paid work and informal caregiving based on their preferences and resources (*capacity*), or whether participation in those activities results from lack of alternatives (*necessity*). The empirical analysis comparing Italy and Korea represents an indirect test of the conceptual model, as SES (wealth and education) proxies the resources and needs of older individuals.

In line with the proposed framework, in Italy – where later-life protection is generous – individuals with higher *capacity* (i.e. in higher-SES groups) are more likely to participate in paid work between ages 50–75. In Korea, where the pension and LTC systems have lagged behind in terms of social protection, women with higher *necessity* (i.e. socioeconomically disadvantaged) are more likely to work, and men in the bottom wealth quintile groups are more likely to provide personal care. The comparison helps contextualise previous research on the association between SES and productive ageing, which finds positive SES gradients in productive participation in some Western countries, and negative gradients (South-) East Asian countries (Arpino & Solé-Auró, 2019; Giang et al., 2019). Interestingly, the SES gradients are most pronounced for women’s employment, which may be due to paid work being less normative among older Italian and Korean women, and therefore more strongly tied to their individual resources and needs.

Contrary to expectations, policy change in Korea between 2006/07 and 2014/15 did not correspond to a reduction of socio-economic differentials in paid work and caregiving. This may reflect the slow maturation of the Korean pension system (Table 1), as the expansion in the coverage of the Korean NPS and Basic Pension Scheme may have not yet expanded social protection for disadvantaged older adults by 2014. As previous research argues (Jeon & Kwon, 2017), the rapid expansion of the Korean LTC system has not necessarily led to more equal access to care services across SES groups, as issues remain with eligibility, care quality, and the coordination between the health and LTC systems (Jeon & Kwon, 2017). As Korea moves towards increased later-life protection, the focus of policy reform should be on maximising *individual capacity* by enabling all older adults to choose whether to engage in paid work and informal care. This is likely to improve not only individual productivity, but also health of older adults, as studies have shown that working and providing care may lead to poor physical and psychological health outcomes if these activities are performed out of obligation rather than choice (Di Gessa et al., 2018; Bom et al., 2019).

In Italy, where not much changed over the study period except the normal age at retirement (Table 1), paid work participation and, to a minor extent, caregiving have remained a prerogative of the wealthier and high-educated. In this context, where older individuals generally benefit from a basic level of economic security, incentives to participate in productive activities should focus more strongly on lower-SES individuals, who tend to have fewer resources facilitating paid work and caregiving. Beneficial measures may include on-the-job retraining, care leave schemes and caregiver relief programmes.

The most important limitation of this study is the inability to test the mechanisms behind the associations between SES and productive roles, which are only suggestive of a potential explanation – that is, later-life social protection – of what drives the observed contextual differences. The conceptual model that the analysis draws upon (Figure 1) assumes that SES and other individual factors ‘determine’ productive engagement by proxying *individual capacity* and *necessity*, but this cannot be tested empirically. Similarly, while the two-country comparison allows for a detailed description of Italy and Korea, I cannot draw conclusions about what it is in each country that shapes differences in the SES distribution of productive activities. Similarly, I am unable to isolate policy changes and study the effect of pension or LTC reforms on the socio-economic distribution of productive activities. Productive ageing is culturally bound (Chen et al., 2016) and so might be its socio-economic distribution. In general, by focussing on later-life social protection, this study leaves out the role of culture as a motive itself behind productive participation (Chen et al., 2016). However, culture alone is unlikely to explain the observed cross-country differences in the SES distribution of productive activities. While paid work may be valued less negatively by those in higher-SES groups due, for instance, to better working conditions, there is no specific reason why this should differ by country. Moreover, while cultural norms around caregiving and family responsibilities vary across countries, Italy and Korea are remarkably similar in their degree of gendered familism in informal care (Saraceno, 2016). At the same time, due to data limitations, these hypotheses remain untested. Empirically, employment and caregiving are negatively related (Van Houtven et al., 2013) and such interdependence may not be adequately addressed by including each outcome as a covariate in the model for the other.

Despite its limitations, this study is among the first to highlight the potential for contextual moderation in the determinants of productive ageing (Teerawichitchainan et al., 2019), and it proposes two mechanisms, *capacity* and *necessity*, as opposite motives for productive participation. Future research on the topic may be oriented towards empirically testing the mechanisms hypothesised here for later-life working and caregiving, as well as other productive activities such as formal volunteering and grandchild care. With respect to those activities, different policies may determine the extent to which individual capacity and necessity prevail as motives behind productive ageing, such as the strength of the non-profit sector for volunteering (Hank, 2011) and public provision of childcare services for grandchild care (Floridi, 2022). Empirically, differences in the SES gradient in productive participation may be studied across many contexts and time points, or around specific policy changes that allow to identify causal effects. Such comparative work may highlight new explanations for the role of socio-economic resources in shaping older adults’ productive participation across contexts.

## Supplemental Material

Supplemental Material - Capacity or Necessity? Comparing the Socio-Economic Distribution of Productive Activities Between Italy and South Korea Click here for additional data file. Supplemental material for Capacity or Necessity? Comparing the Socio-Economic Distribution of Productive Activities Between Italy and South Korea by Ginevra Floridi in Research on Aging

## Note

1. ADLs include dressing, walking across a room, bathing or showering, eating, getting in and out of bed, and using the toilet. IADLs include using a map, preparing a hot meal, shopping for groceries, making telephone calls, taking medications, doing work around the house or garden, and managing money.
