# Supplementary material for: Capacity or Necessity? Comparing the Socio-Economic Distribution of Productive Activities Between Italy and South Korea
Source: Res Aging. 2022 Apr 23;45(1):21–34. doi: 10.1177/01640275221089203 (PMC9814022; doi:10.1177/01640275221089203)
Supplement: Supplemental Material - Capacity or Necessity? Comparing the Socio-Economic Distribution of Productive Activities Between Italy and South Korea [file sj-pdf-1-roa-10.1177_01640275221089203.pdf]

**Supplementary Tables and Figures**

**Supplementary table 1.** Coding of all variables in the study

| **Outcome variables** |  |
| --- | --- |
| Work 30+ hours | 1 = working (employed, self-employed, or for family business) for at least 30 hours/week ; 0 = otherwise |
| Informal care provision | 1 = providing personal care with ADL or IADL limitations to a family member or friend living within or outside the household ; 0 = otherwise |
| **Independent variables** |  |
| Education | 0 = “low” (none or up to elementary schooling) ; 1 = “high” (secondary or higher schooling) |
| Wealth quintile group | 1 = bottom 20% ; 2 = 20-40^th^ percentile ; 3 = 40-60^th^ percentile ; 4 = 60-80^th^ percentile ; 5 = top 20%  Percentiles obtained within groups defined by country and wave, from equivalised wealth (including financial and real assets, minus debt). |
| Age | In years (min = 50, max = 75) |
| Marital status | 1 = married ; 2 = widowed ; 3 = non-married (never married, separated, and divorced) |
| Rural (vs urban) area | 0 = living in an urban area (town or city) ; 1 = living in a rural area or village |
| Any ADL limitations | 0 = no limitations with ADLs ; 1 = at least one limitation in ADLs |
| Any IADL limitations | 0 = no limitations with IADLs ; 1 = at least one limitation in IADLs |
| Self-rated health | 1 = excellent ; 2 = very good ; 3 = good ; 4 = fair ; 5 = poor |
| Cognition score | Interval-level score from 0 to 4, where 0 indicates no recollection of day of the week, date, month, and year, and 4 indicates correct identification of all four items. |
| Parent status | 0 = childless ; 1 = has children, all children living outside the household ; 2 = has children, and at least one child is co-resident |
| Either parent alive | 0 = neither parent is still alive ; 1 = at least one parent is still alive |
| Any grandchildren | 0 = no grandchildren ; 1 = respondent has at least one grandchild |
| Giving financial support | 0 = in the past 12 months, respondent did not give any financial support to family members or friends above 250 Euros (or the PPP-equivalent for that year in Korean won) ; 1= in the past 12 months, respondent gave financial support to family members or friends above 250 Euros (or the PPP-equivalent for that year in Korean won) |
| Receiving financial support | 0 = in the past 12 months, respondent did not receive any financial support from family members or friends above 250 Euros (or the PPP-equivalent for that year in Korean won) ; 1= in the past 12 months, respondent received financial support from family members or friends above 250 Euros (or the PPP-equivalent for that year in Korean won) |
| **Auxiliary variables** |  |
| Gender | 1 = male ; 2 = female (identified as sex) |
| Country | 0 = Italy ; 1 = Korea |
| Wave | 0 = 2006/7 (SHARE wave 2 or KLoSA wave 1) ; 1 = 2014/15 (SHARE wave 6 or KLoSA wave 5) |

**Supplementary table 2**. Preliminary analysis 1: statistical significance of interaction coefficients between each independent variable and the country indicator (Italy vs. Korea) in separate models by observation period and gender. Tick (✓) indicates statistical significance (p<0.05).

|  | **Outcome: paid work for 30+ hours/week** | | | | **Outcome: informal caregiving for sick or disabled adults** | | | |
| --- | --- | --- | --- | --- | --- | --- | --- | --- |
|  | **2006/07** | | **2014/15** | | **2006/07** | | **2014/15** | |
|  | **Men** | **Women** | **Men** | **Women** | **Men** | **Women** | **Men** | **Women** |
| Education * country | ✓ | ✓ |  | ✓ |  |  |  |  |
| Wealth quintile group * country | ✓ | ✓ |  | ✓ | ✓ | ✓ |  |  |
| Age * country | ✓ | ✓ | ✓ | ✓ | ✓ |  | ✓ |  |
| Marital status * country | ✓ |  |  |  |  |  |  |  |
| Rural (vs urban) area * country | ✓ | ✓ | ✓ | ✓ |  |  |  |  |
| Any ADL limitations * country |  |  | ✓ |  |  |  |  |  |
| Any IADL limitations * country |  | ✓ | ✓ |  | ✓ |  |  | ✓ |
| Self-rated health * country | ✓ |  |  |  | ✓ |  |  |  |
| Cognition score * country | ✓ |  | ✓ | ✓ | ✓ |  |  |  |
| Parent status * country |  |  |  | ✓ |  |  |  |  |
| Either parent alive * country |  |  |  |  | ✓ |  | ✓ |  |
| Any grandchildren * country |  | ✓ |  | ✓ |  |  |  |  |
| Giving fin. support * country | ✓ |  |  |  | ✓ |  | ✓ | ✓ |
| Receiving fin. support * country |  |  | ✓ | ✓ |  | ✓ |  |  |
| Informal care provision * country | ✓ | ✓ |  |  | n/a | n/a | n/a | n/a |
| Full-time work * country | n/a | n/a | n/a | n/a | ✓ |  |  |  |
| N. individuals | 3,975 | 4,588 | 4,138 | 5,069 | 3,753 | 4,588 | 4,034 | 5,064 |

**Supplementary table 3**. Preliminary analysis 2: statistical significance of interaction coefficients between each independent variable and observation period indicator (2006/07 vs. 2014/15) in separate models by country and gender. Tick (✓) indicates statistical significance (p<0.05).

|  | **Outcome: paid work for 30+ hours/week** | | | | **Outcome: informal caregiving for sick or disabled adults** | | | |
| --- | --- | --- | --- | --- | --- | --- | --- | --- |
|  | **Italy** | | **Korea** | | **Italy** | | **Korea** | |
|  | **Men** | **Women** | **Men** | **Women** | **Men** | **Women** | **Men** | **Women** |
| Education * time |  | ✓ |  |  |  |  |  |  |
| Wealth quintile group * time | ✓ |  | ✓ |  |  | ✓ |  |  |
| Age * time | ✓ |  | ✓ |  |  | ✓ |  |  |
| Marital status * time |  |  |  |  |  |  |  |  |
| Rural (vs urban) area * time |  |  |  |  | ✓ |  |  |  |
| Any ADL limitations * time |  |  |  |  |  |  |  |  |
| Any IADL limitations * time |  |  |  | ✓ |  |  |  | ✓ |
| Self-rated health * time |  |  |  |  |  |  |  |  |
| Cognition score * time | ✓ |  | ✓ |  |  |  |  |  |
| Parent status * time |  |  |  |  |  |  |  |  |
| Either parent alive * time |  |  |  |  |  | ✓ |  |  |
| Any grandchildren * time |  |  |  | ✓ |  |  |  |  |
| Giving fin. support * time |  |  |  |  |  |  |  | ✓ |
| Receiving fin. support * time |  |  |  |  |  |  |  |  |
| Informal care provision * time |  |  |  |  |  |  |  |  |
| Full-time work * time |  |  |  |  |  |  |  |  |
| N. individuals | 2,586 | 3,128 | 5,527 | 6,529 | 2,580 | 3,120 | 5,207 | 6,532 |

**Supplementary table 4**. Coefficients from logistic regression models of paid work on full set of covariates, separately by country and gender

|  | **Italian men** | **Korean men** | **Italian women** | **Korean women** |
| --- | --- | --- | --- | --- |
|  |  |  |  |  |
| 2014/15 (vs. 2006/07) | 4.345 (1.466) ** | 3.595 (0.723) *** | -2.368 (1.707) | 2.377 (0.694) *** |
| High education (vs. low) | 0.276 (0.225) | -0.057 (0.099) | 0.713 (0.273) ** | -0.315 (0.108) ** |
| High education*2014/15 | -0.178 (0.282) | 0.045 (0.164) | -0.144 (0.325) | 0.011 (0.152) |
| Wealth quint.: 1^st^ (ref) |  |  |  |  |
| 2^nd^ | -0.395 (0.299) | 0.255 (0.142) ~ | -0.088 (0.399) | 0.123 (0.137) |
| 3^rd^ | -0.722 (0.309) * | 0.180 (0.138) | 0.373 (0.385) | -0.051 (0.139) |
| 4^th^ | -0.612 (0.307) * | 0.033 (0.138) | 0.094 (0.379) | -0.225 (0.146) |
| 5^th^ | -0.417 (0.321) | 0.027 (0.139) | 0.433 (0.386) | -0.458 (0.158) ** |
| Wealth quint*2014/15 |  |  |  |  |
| 2^nd^ * 2014/15 | 0.912 (0.375) * | 0.381 (0.232) ~ | 0.467 (0.455) | -0.101 (0.191) |
| 3^rd^ * 2014/15 | 1.326 (0.389) *** | 0.316 (0.334) | 0.407 (0.441) | 0.055 (0.192) |
| 4^th^ * 2014/15 | 1.159 (0.386) ** | 0.309 (0.222) | 0.638 (0.438) | 0.195 (0.196) |
| 5^th^ * 2014/15 | 1.204 (0.399) ** | 0.197 (0.222) | 0.421 (0.444) | -0.006 (0.207) |
| Age | -0.201 (0.019) *** | -0.110 (0.008) *** | -0.229 (0.027) *** | -0.105 (0.008) *** |
| Age*2014/15 | -0.074 (0.024) ** | -0.046 (0.011) *** | 0.051 (0.029) ~ | -0.024 (0.011) * |
| Marital status (ref: married) |  |  |  |  |
| Widowed | -0.292 (0.529) | -0.294 (0.186) | -0.428 (0.307) | 0.222 (0.090) * |
| Never mar., sep., divorced | -0.275 (0.239) | -0.284 (0.197) | 0.604 (0.190) *** | 0.982 (0.175) *** |
| Rural (vs. urban) | -0.085 (0.124) | 0.787 (0.082) *** | -0.184 (0.130) | 0.785 (0.075) *** |
| 1+ ADL limitations (vs. no) | -0.432 (0.431) | -1.510 (0.380) *** | -0.175 (0.375) | -1.819 (0.618) *** |
| 1+ IADL limitations (vs.no) | -0.963 (0.392) * | -0.071 (0.110) | -0.416 (0.329) | 0.258 (0.186) |
| SR health (ref: excellent) |  |  |  |  |
| Very good | 0.262 (0.224) | -0.109 (0.202) | -0.019 (0.228) | 0.449 (0.273) ~ |
| Good | 0.095 (0.199) | -0.447 (0.201) * | -0.088 (0.212) | 0.225 (0.270) |
| Fair | -0.454 (0.223) * | -0.777 (0.207) *** | -0.334 (0.227) | 0.173 (0.272) |
| Poor | -1.344 (0.409) *** | -2.080 (0.249) *** | -0.502 (0.372) | -0.208 (0.291) |
| Cognition score | 0.036 (0.185) | 0.105 (0.065) | 0.401 (0.214) ~ | 0.026 (0.055) |
| Parent status (ref: childless) |  |  |  |  |
| All children outside hh | 0.137 (0.248) | 0.615 (0.252) * | -0.054 (0.218) | 0.986 (0.252) *** |
| Coresident children | 0.327 (0.239) | 0.690 (0.249) ** | -0.238 (0.205) | 0.910 (0.249) *** |
| Either parent alive (vs. no) | 0.134 (0.129) | 0.042 (0.079) | -0.057 (0.125) | -0.194 (0.075) ** |
| Any grandchildren (vs. no) | -0.227 (0.139) | -0.006 (0.088) | -0.559 (0.141) *** | -0.026 (0.078) |
| Giving fin. trans. (vs. no) | 0.116 (0.127) | 0.414 (0.079) *** | 0.392 (0.127) ** | 0.260 (0.078) *** |
| Receiving fin. trans. (vs.no) | -0.607 (0.234) ** | -0.117 (0.075) | -0.148 (0.212) | -0.136 (0.073) ~ |
| Informal caregiving (vs. no) | -0.172 (0.143) | -0.683 (0.165) *** | -0.012 (0.132) | -0.850 (0.146) *** |
|  |  |  |  |  |
| n. observations | 2586 | 5527 | 3128 | 6555 |
| Pseudo R-squared | 0.378 | 0.228 | 0.289 | 0.146 |

***, **, *, ~ : p<0.001, p<0.01, p<0.05, p<0.10

**Supplementary table 5**. Coefficients from logistic regression models of caregiving for sick or disabled adults on full set of covariates, separately by country and gender.

|  | **Italian men** | **Korean men** | **Italian women** | **Korean women** |
| --- | --- | --- | --- | --- |
|  |  |  |  |  |
| 2014/15 (vs. 2006/07) | -1.676 (1.382) | -4.934 (2.437) * | -1.865 (0.979) ~ | -3.189 (1.803) ~ |
| High education (vs. low) | 0.263 (0.232) | 0.310 (0.303) | -0.035 (0.165) | 0.346 (0.224) |
| High education*2014/15 | 0.446 (0.319) | 0.080 (0.558) | 0.010 (0.013) | -0.109 (0.416) |
| Wealth quint.: 1^st^ (ref) |  |  |  |  |
| 2^nd^ | 0.057 (0.350) | -1.001 (0.402) * | 0.169 (0.245) | 0.062 (0.267) |
| 3^rd^ | 0.260 (0.345) | -0.909 (0.379) * | 0.475 (0.243) ~ | -0.282 (0.291) |
| 4^th^ | 0.235 (0.347) | -1.025 (0.374) ** | 0.125 (0.252) | -0.293 (0.300) |
| 5^th^ | 0.472 (0.349) | -1.202 (0.387) ** | 0.537 (0.244) * | -0.303 (0.317) |
| Wealth quint*2014/15 |  |  |  |  |
| 2^nd^ * 2014/15 | 0.071 (0.442) | 0.437 (0.763) | -0.164 (0.310) | -0.796 (0.565) |
| 3^rd^ * 2014/15 | -0.250 (0.446) | 0.563 (0.713) | -0.626 (0.312) * | -0.182 (0.539) |
| 4^th^ * 2014/15 | -0.376 (0.450) | 0.731 (0.701) | -0.182 (0.319) | 0.118 (0.512) |
| 5^th^ * 2014/15 | -0.321 (0.441) | 0.852 (0.704) | -0.520 (0.311) ~ | 0.261 (0.510) |
| Age | 0.003 (0.019) | 0.092 (0.026) *** | -0.001 (0.013) | 0.011 (0.017) |
| Age*2014/15 | 0.019 (0.020) | 0.060 (0.035) ~ | 0.030 (0.015) * | 0.038 (0.026) |
| Marital status (ref: married) |  |  |  |  |
| Widowed | -0.877 (0.605) | 0.165 (0.489) | -0.444 (0.175) * | -0.641 (0.238) ** |
| Never mar., sep., divorced | -0.121 (0.297) | -0.207 (0.751) | -0.177 (0.202) | -0.113 (0.409) |
| Rural (vs. urban) | -0.021 (0.138) | -0.092 (0.261) | 0.061 (0.099) | -0.361 (0.215) ~ |
| 1+ ADL limitations (vs. no) | 1.126 (0.297) *** | -0.099 (1.121) | 0.324 (0.209) | -0.835 (0.665) |
| 1+ IADL limitations (vs.no) | 0.240 (0.294) | -0.488 (0.428) | 0.103 (0.168) | 0.553 (0.334) ~ |
| SR health (ref: excellent) |  |  |  |  |
| Very good | 0.061 (0.272) | -0.803 (0.445) ~ | -0.279 (0.208) | 1.003 (1.025) |
| Good | 0.088 (0.243) | -1.156 (0.453) * | -0.171 (0.188) | 1.231 (1.021) |
| Fair | 0.121 (0.260) | -1.335 (0.484) ** | -0.193 (0.194) | 1.578 (1.021) |
| Poor | -0.569 (0.411) | -1.777 (0.690) ** | -0.045 (0.252) | 1.503 (1.048) |
| Cognition score | 0.410 (0.199) * | 0.011 (0.208) | -0.029 (0.104) | -0.201 (0.101) |
| Parent status (ref: childless) |  | (empty) |  |  |
| All children outside hh | -0.207 (0.306) | -0.272 (0.234) | 0.145 (0.226) | -0.687 (0.452) |
| Coresident children | 0.197 (0.296) | (ref.) | 0.470 (0.219) * | -0.391 (0.435) |
| Either parent alive (vs. no) | 0.695 (0.157) *** | 2.323 (0.251) *** | 1.077 (0.111) *** | 0.471 (0.190) * |
| Any grandchildren (vs. no) | -0.166 (0.154) | 0.095 (0.290) | -0.037 (0.112) | -0.116 (0.222) |
| Giving fin. trans. (vs. no) | 0.411 (0.137) ** | -0.715 (0.268) ** | 0.374 (0.101) *** | -0.183 (0.215) |
| Receiving fin. trans. (vs.no) | 0.324 (0.228) | 0.156 (0.239) | 0.365 (0.163) * | -0.073 (0.182) |
| Paid work 30+hrs (vs. no) | -0.490 (0.186) ** | -0.319 (0.232) | 0.093 (0.131) | 0.090 (0.186) |
|  |  |  |  |  |
| n. observations | 2580 | 5406 | 3120 | 6555 |
| Pseudo R-squared | 0.058 | 0.135 | 0.065 | 0.048 |

***, **, *, ~ : p<0.001, p<0.01, p<0.05, p<0.10

**Supplementary figure 1**. The “productivity in later life” model

**
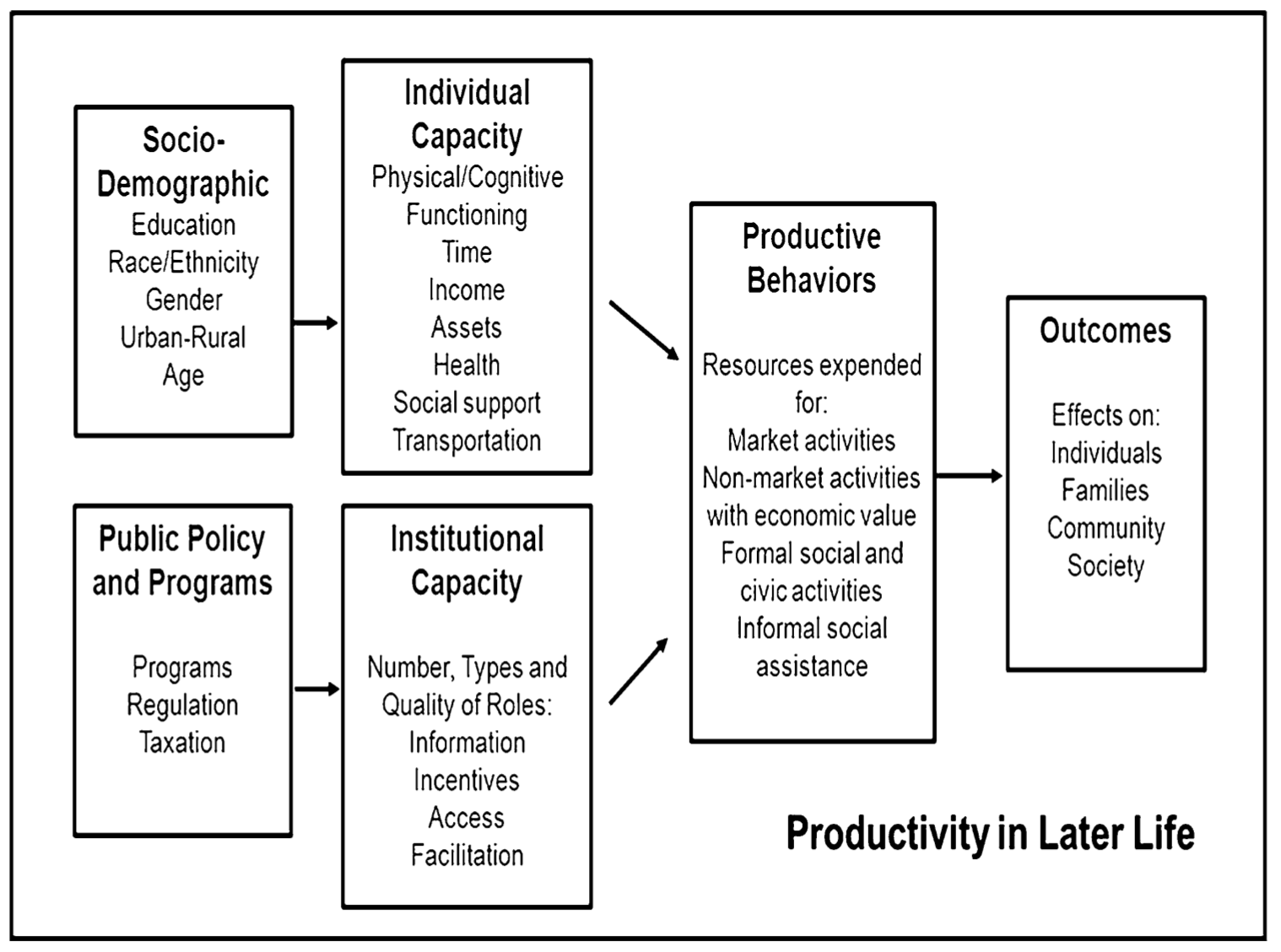
**

Source: Sherraden et al. (2001)

**Supplementary figure 2.** Average predicted probability of full-time paid work with 95% confidence interval, calculated at different quintile groups of equivalised wealth*, by gender. Calibrated cross-sectional survey weights used as probability weights.

* The values of all other covariates in the model are fixed at their mean values.

**Supplementary figure 3.** Average predicted probability of full-time paid work with 95% confidence interval, calculated at different levels of educational attainment*, by gender. Calibrated cross-sectional survey weights used as probability weights.

* The values of all other covariates in the model are fixed at their mean values.

**Supplementary figure 4.** Average predicted probability of caregiving for sick or disabled adults with 95% confidence interval, calculated at different quintile groups of equivalised wealth*, by gender. Calibrated cross-sectional survey weights used as probability weights.

* The values of all other covariates in the model are fixed at their mean values.

**Supplementary figure 5.** Average predicted probability of caregiving for sick or disabled adults with 95% confidence interval, calculated at different levels of educational attainment*, by gender. Calibrated cross-sectional survey weights used as probability weights.

* The values of all other covariates in the model are fixed at their mean values.
